# Supplementary figures and images for: Change the direction: 3D optimal control simulation by directly tracking marker and ground reaction force data
Source: PeerJ. 2023 Feb 7;11:e14852. doi: 10.7717/peerj.14852 (PMC9912948; doi:10.7717/peerj.14852)

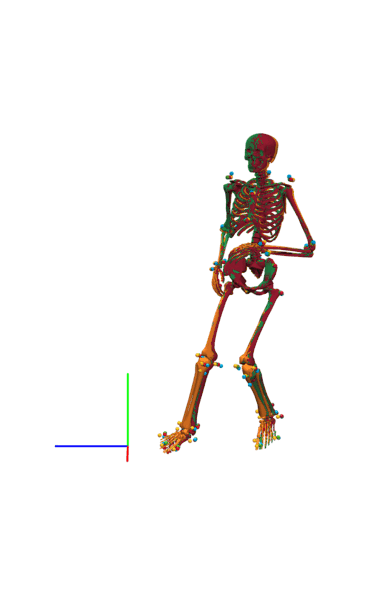

Supplement: Supplemental Information 1 — The result of inverse kinematics, coordinate tracking, and marker tracking is represented in red, orange, and green, respectively. The measured marker positions are displayed in blue. The motion had in total 149 samples at 175 Hz. [file peerj-11-14852-s001.gif]

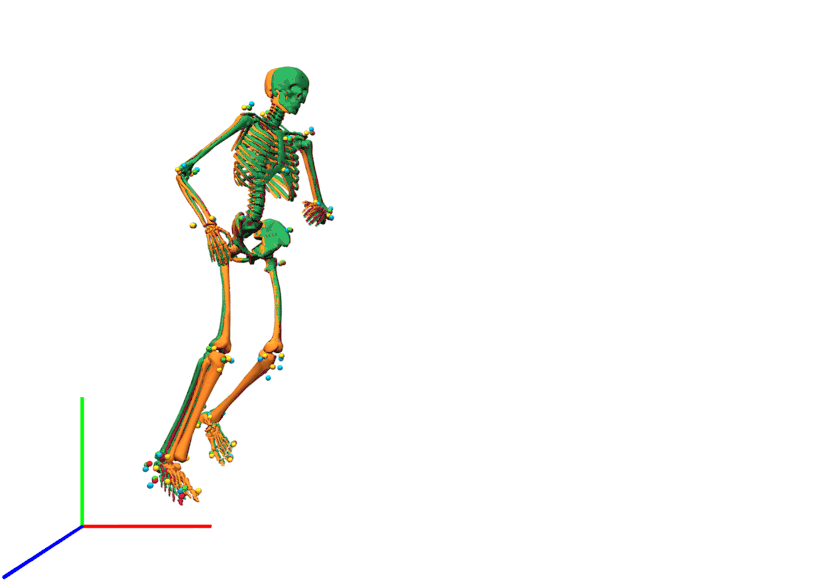

Supplement: Supplemental Information 2 — The result of inverse kinematics, coordinate tracking, and marker tracking is represented in red, orange, and green, respectively. The measured marker positions are displayed in blue. The motion had in total 149 samples at 175 Hz. [file peerj-11-14852-s002.gif]
